# Quality of life, depression and sexual function in men living with HIV under effective antiretroviral therapy compared with healthy controls

**DOI:** 10.1080/1179271X.2026.2664443

**Published:** 2026-05-08

**Authors:** Özlem Güler, Demir Akhan, Gamze Kara Sözen, Sıla Akhan

**Affiliations:** ^a^ Department of Infectious Diseases and Clinical Microbiology, Kocaeli University, School of Medicine, Kocaeli, Turkey; ^b^ Independent Researcher, Kocaeli, Turkey

**Keywords:** cART, sustained virologic response, health surveys, psychological wellbeing, sexual health

## Abstract

**Purpose:**

Combination antiretroviral therapy (cART) has improved outcomes in individuals living with HIV. This study investigated quality of life, mental health, and sexual function in virologically suppressed men.

**Patients and methods:**

This case-control study compared quality of life, depression, and sexual function of 39 heterosexual men on cART with viral suppression and 60 healthy male controls. Participants completed the EUROHIS-QOL 8, Beck Depression Inventory (BDI), and Arizona Sexual Experience Scale (ASEX) questionnaires.

**Results:**

HIV group’s mean age was 39 years, controls 41.5 years, with similar demographics. Median EUROHIS-QOL8 and BDI scores were similar between groups. While ASEX scores were higher in HIV patients (*p* = .032), this difference did not result in a higher prevalence of sexual dysfunction based on established ASEX criteria. BDI scores strongly negatively correlated with EUROHIS-QOL8 in the HIV group (*r* = −.731, *p* < .001) and moderately in controls (*r* = −.475, *p* < .001). Quality of life decreased with depressive symptoms in both groups.

**Conclusion:**

Among men with HIV who achieved viral suppression, no significant differences were found versus healthy controls in quality of life, depression, or sexual dysfunction. These findings support individual evaluation rather than HIV status-based assumptions.

## GRAPHICAL ABSTRACT

**Figure:**
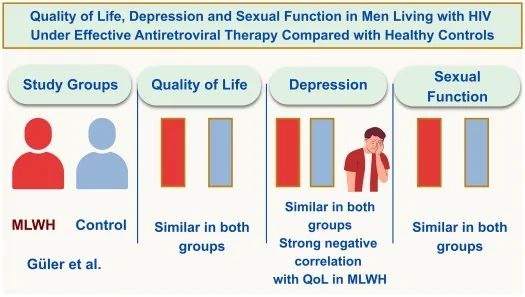

## 1. Introduction

Effective combination antiretroviral therapy (cART) has extended the life expectancy of individuals living with HIV and significantly improved the clinical course of the disease. However, significant problems may persist in terms of quality of life, mental health, and sexual function, even with long-term cART.

The World Health Organization defines quality of life as an individual’s perception of their overall position in life, shaped by the cultural context and value systems surrounding them, and influenced by their personal goals, expectations, and concerns.^1^ Although cART has been shown to improve quality of life, long-term users may experience fatigue, insomnia, changes in physical appearance, and medication side effects. These issues can lead to a decline in quality of life and depression.^2–5^ Additionally, the stigma associated with HIV directly impacts life satisfaction and mental health negatively.^6^ Sexual dysfunction is commonly reported among individuals living with HIV. Studies have identified erectile dysfunction, premature ejaculation, and low sexual desire in individuals using long-term cART.^7^

This study aimed to compare the quality of life, level of depression, and sexual function of heterosexual men living with virologically suppressed HIV under cART with those of healthy male controls matched by age and basic demographic characteristics. In addition to the primary comparison, the objective of this study was to reveal the correlations between quality of life, depressive symptoms, and sexual function within each group.

## 2. Material and methods

### 2.1. Study design and setting

This comparative, case-control, observational study was conducted at the outpatient clinic of the Department of Infectious Diseases and Clinical Microbiology at a tertiary care hospital. Two interviewers collected data face-to-face between 26 September and 17 October 2025. This study was reported in accordance with the Strengthening the Reporting of Observational Studies in Epidemiology (STROBE) Statement checklist for cross-sectional studies, as recommended by the EQUATOR Network.^8^ The research was conducted in accordance with the principles of the World Medical Association Declaration of Helsinki (2013 revision) and the Non-Interventional Clinical Research Ethics Committee of the Kocaeli University Faculty of Medicine protocols. The project was registered under number 2025/450 and received approval under code GOKAEK-2025/19/06 on 25 September 2025. Since the study was survey-based, verbal informed consent was obtained from the participants who agreed to participate. This observational, non-interventional study was retrospectively registered at ClinicalTrials.gov (NCT07376239) on 21 January 2026.

### 2.2. Case definitions and exclusion and inclusion criteria

The study group consisted of heterosexual men living with HIV (MLWH) who were receiving cART and had achieved virological suppression, confirmed at least at two separate visits. The control group comprised volunteers among the companions of patients attending the same clinic for other medical reasons, who were matched for age and basic demographic characteristics. Controls were recruited from among the companions of patients attending the clinic for non-debilitating conditions. They were not recruited from among caregivers of severely ill individuals. The inclusion criteria were as follows: age ≥ 18 years, male sex, regular ART use, and virologic suppression confirmed at two consecutive visits. The exclusion criteria included advanced HIV disease, female sex, any comorbidity, and cognitive or communication difficulties that could interfere with completing the survey.

### 2.3. Data collection tools and process

During the three-week study period, questionnaires were administered to all eligible patients attending the clinic, and a similar number of controls matched for age and key demographic characteristics were recruited on a voluntary basis. No formal sample size calculation was performed because the goal was to include all accessible populations within the defined period. Sociodemographic data, including age, educational status, marital status, regular physical activity, living alone or with family, history of smoking and alcohol use and antiretroviral therapy were recorded. Mental status was assessed using the Beck Depression Inventory (BDI), sexual function using the Arizona Sexual Experience Scale (ASEX), and quality of life using the European Health Interview Survey [EUROHIS-QOL 8 (WHOQOL-8)] scale. Validated Turkish versions of these instruments were used. Participants completed all the questionnaires anonymously in a quiet environment without researcher intervention.

The BDI is a widely used self-report scale that assesses the severity of depression. It consists of 21 items, each rated on a four-point scale ranging from 0 to 3. In Turkey, a threshold of 17 is commonly used to identify cases. Accordingly, total scores are interpreted as follows: 0–9: no depressive symptoms; 10–16: minimal symptoms; 17–29: moderate symptoms; and 30–63: severe symptoms.^9^ The Turkish validation study reported a Cronbach’s α of 0.80.^10^

The ASEX is a brief, concise, five-item self-report scale developed to assess sexual dysfunction. The scale measures the core areas of sexual function: sexual desire, sexual arousal, vaginal lubrication or penile erection, achieving orgasm, and satisfaction from orgasm. Each item is scored from one to six, with a total score ranging from five to 30. A total score of 19 or higher on the ASEX, a score of 5 or higher on any single item, or a score of 4 or higher on at least three items is considered an indication of sexual dysfunction.^11^ Furthermore, ASEX has been validated through multi-center studies as being valid and reliable in different cultures and languages, including Turkish.^12^

The EUROHIS-QOL-8 is an eight-item, five-point Likert scale. Each item is scored on a scale of 1 and 5. A higher score indicates a better quality of life.^13^ The mean item score for each participant was calculated by dividing the total score by eight.^14^ In the primary analyses, the score was treated as a continuous variable, and the results were reported as the mean ± standard deviation (SD) or median [interquartile range (IQR)]. In exploratory secondary analyses, scores were classified as ≥4.00 “high,” 3.00–3.99 “moderate,” and <3.00 “low,” consistent with the operational approach in population surveillance literature.^15^ These thresholds are not clinical cut-offs and were used solely to facilitate visualization and subgroup comparisons.

### 2.4. Statistical analyses

Statistical analyses were performed using IBM SPSS Statistics version 29.0 (IBM Corp., Armonk, NY, USA; institutional license at Kocaeli University). Distributional assumptions were assessed using the Kolmogorov–Smirnov or Shapiro–Wilk tests. Continuous variables were summarized as mean ± standard deviation for normally distributed data or as median with IQR otherwise. Categorical variables were summarized as numbers and percentages. As continuous variables did not meet the normality assumptions, between-group comparisons were conducted using the Mann–Whitney U test. Categorical variables were compared using the chi-square test or Fisher’s exact test as appropriate. The correlations between continuous variables were assessed using Spearman’s rank correlation coefficient (r). Separate analyses were performed for MLWH and controls to evaluate the relationships between age, depression (BDI scores), quality of life (EUROHIS-QOL8 scores), and sexual function (ASEX scores). The magnitude of association was interpreted using |r| with a priori cut-offs: 0.00–0.10 negligible, 0.10–0.39 weak, 0.40–0.69 moderate, 0.70–0.89 strong, and 0.90–1.00 very strong.^16^ All tests were two-sided with *p* < .05 considered statistically significant. Effect sizes are reported as r for Mann–Whitney *U* tests and as φ (phi) or Cramer’s *V* for chi-square tests. Effect size magnitudes were interpreted according to conventional criteria: small (0.10–0.29), medium (0.30–0.49), and large (≥0.50).

## 3. Results

### 3.1. Baseline characteristics

**Figure 1.**
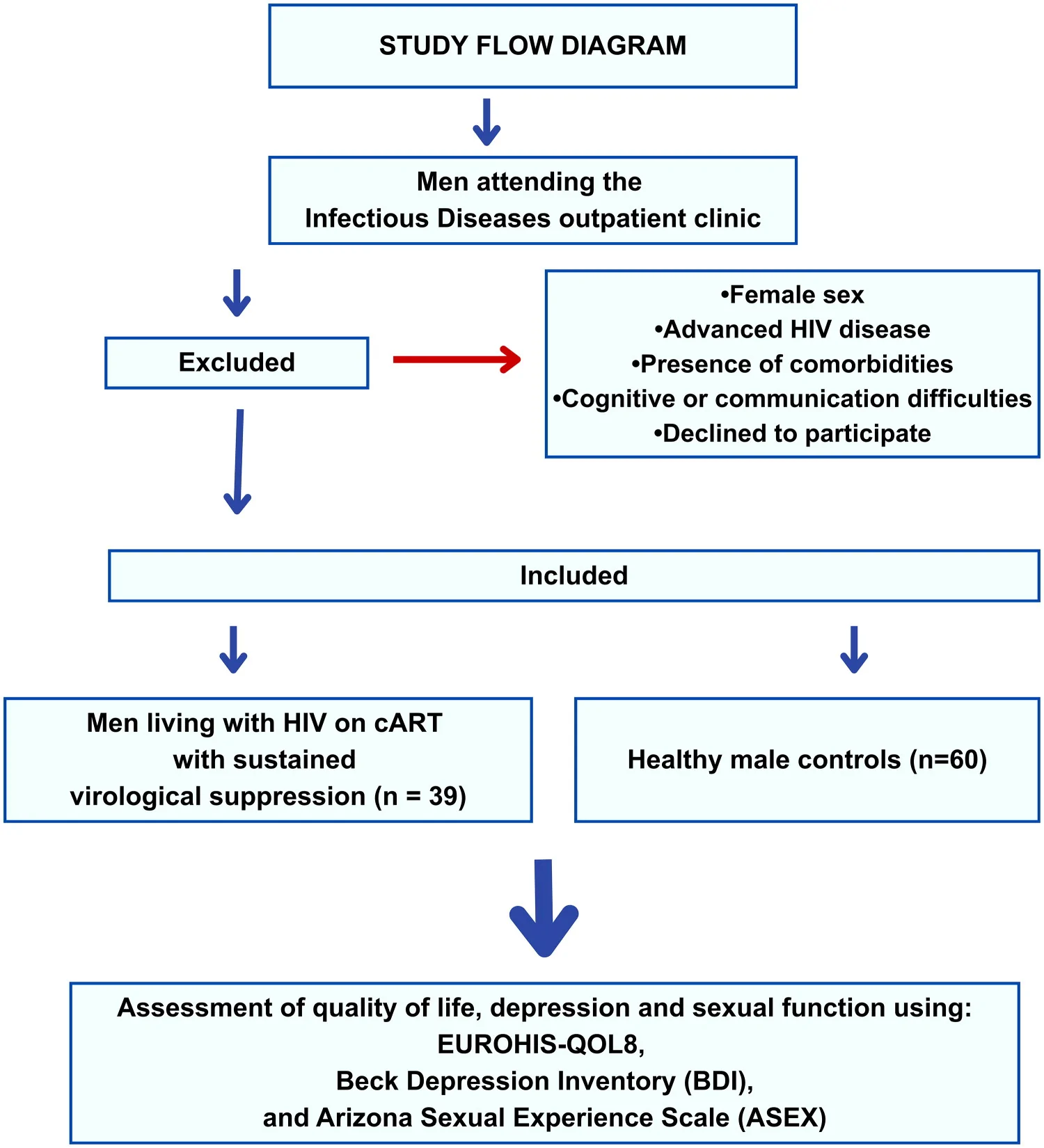
Study flow diagram.

The sample population of this study included 99 participants: 39 MLWH and 60 healthy controls. The participant selection process and final study population are summarized in Figure 1. The median age was 39 (IQR: 29–46) years in the HIV group and 41.5 (IQR: 24–48) years in the control group (*p* = .688). The demographic characteristics, including educational level, marital status, living circumstances, regular exercise habits, alcohol intake, and smoking status, were homogeneous across the study groups (Table 1). Current smoking was more prevalent among MLWH (71.8% vs. 50.0%); however, the difference was not statistically significant (*p* = .052).

**Table 1.** Group comparisons of baseline characteristics.

Variable	MLWH, *n* = 39	Control, *n* = 60	*p* Value	*r*, ϕ, Cramer’s *V*
Age, median (IQR)	39 (29–46)	41.5 (24–48)	.688^a^	**0.040**
**Educational level, *n* (%)**			.892^b^	**0.106**
Non-university	24 (61.5)	39 (65)		
University graduate	15 (38.5)	21 (35)		
**Marital status, *n* (%)**			.421^b^	**0.143**
Single	24 (61.5)	33 (55)		
Divorced	4 (10.3)	3 (5)		
Married	11 (28.2)	24 (40)		
**Single household, *n* (%)**	23 (59)	23 (38.3)	.071^b^	**0.202**
**Regular exercise, *n* (%)**	16 (41)	22 (36.7)	.823^b^	**0.044**
**Alcohol intake, *n* (%)**	15 (38.5)	17 (28.3)	.405^b^	**0.106**
**Current smoker, *n* (%)**	28 (71.8)	30 (50)	.052^b^	**0.216**
**Treatment**				
** Two NRTIs + INSTI, *n* (%)**	**31 (79.5)**	**N/A**		
** One NRTI + INSTI, *n* (%)**	**8 (20.5)**	**N/A**		

Abbreviations: MLWH, men living with HIV; *r*: effect size for Mann–Whitney *U* test; φ (phi) or Cramer’s *V*, effect size for chi-square tests; NRTI, nucleoside reverse transcriptase inhibitors; INSTI, integrase strand transfer inhibitor; N/A, not applicable.

^a^Mann–Whitney *U* test.

^b^Chi-square test.

Bold values indicate statistically significant correlations (*p* < .05).

### 3.2. Quality of life, depression, and sexual dysfunction

**Figure 2.**
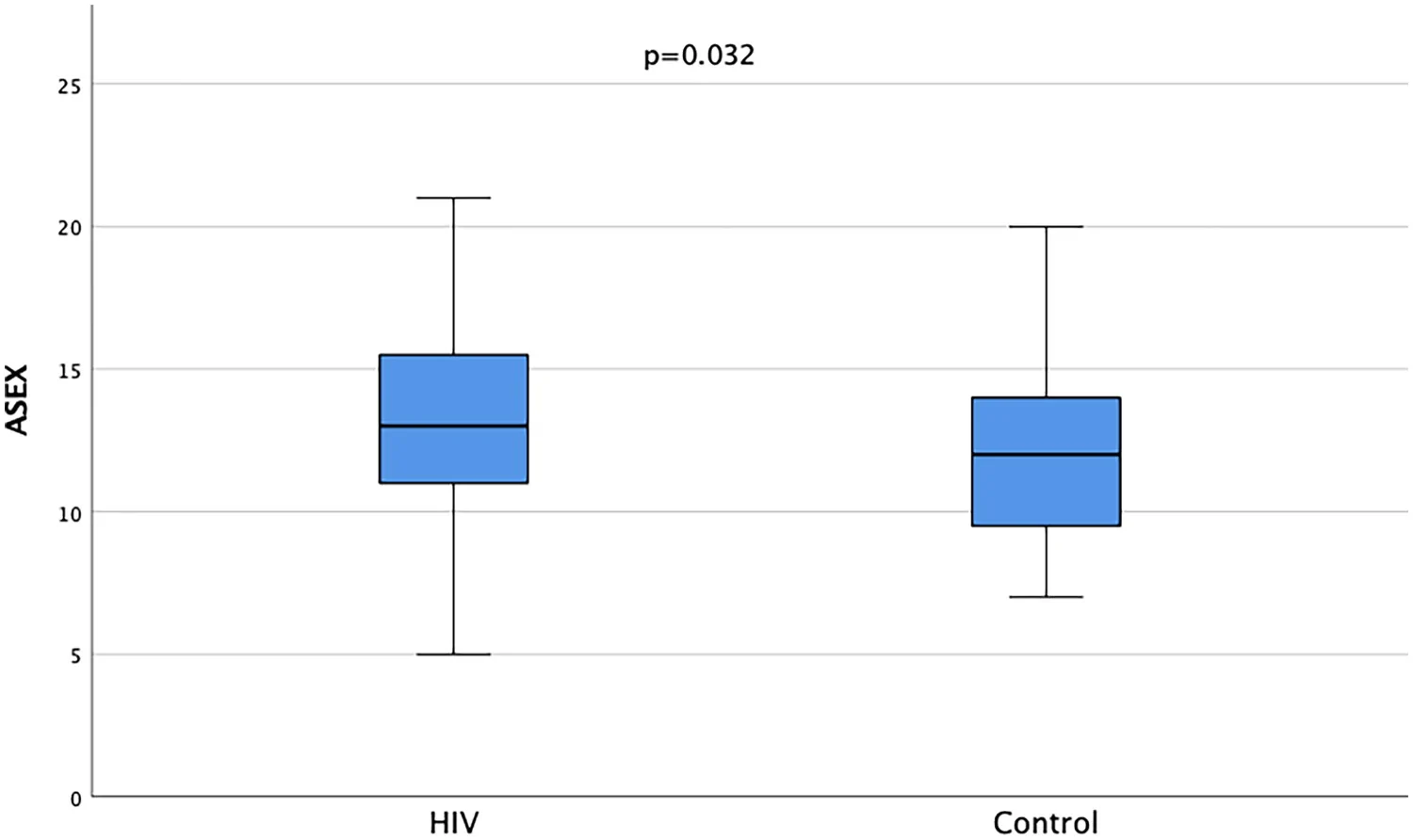
Boxplot comparison of ASEX scores between men living with HIV and the controls. The boxes show the interquartile range, and the line within each box represents the median.

The median EUROHIS-QOL8 scores, which reflect quality of life, were similar between the two groups [3.75 (IQR: 3–4.13) and 3.88 (IQR: 3.16–4.22), *p* = .568]. The severity of depression, as measured by the BDI, did not differ significantly [12 (IQR: 5–18) vs. 9 (IQR: 4–17.75); *p* = .364]. However, the results of the ASEX, a measure of sexual function, indicated higher scores in the HIV group [13 (IQR: 11–16) vs. 12 (IQR: 9.25–14); *p* = .032]. This finding indicates a modest difference in sexual function scores between the groups, rather than a difference in the prevalence of sexual dysfunction (Table 2, Figure 2). Higher BDI and ASEX scores indicate worse depression and greater sexual dysfunction, respectively, and higher EUROHIS-QOL8 scores indicate better quality of life. Categorical analyses revealed that the distributions of quality of life levels (low, moderate, and high), BDI-based depression severity (none, minimal, moderate, and severe), and ASEX-based sexual dysfunction (present and absent) were similar across groups (Table 2, *p* > .05).

**Table 2.** Group comparisons of quality of life, depression, and sexual function.

Variable	MLWH, *n* = 39	Control, *n* = 60	*p* Value	*r*, ϕ, Cramer’s *V*
**EUROHIS-QOL8, median (IQR)**	3.75 (3–4.13)	3.88 (3.16–4.22)	.568^a^	**0.057**
**BDI, median (IQR)**	12 (5–18)	9 (4–17.75)	.364^a^	**0.091**
**ASEX, median (IQR)**	13 (11–16)	12 (9.25–14)	.032^a^	**0.215**
**EUROHIS-QOL8–assessed quality of life, *n* (%)**				**0.060**
Low quality of life	6 (15.4)	9 (15)		
Moderate quality of life	18 (46.2)	23 (38.3)	.723^b^	
High quality of life	15 (38.5)	28 (46.7)		
**BDI-assessed depression severity, *n* (%)**			.642^b^	
No depression	9 (23.1)	13 (21.7)		
Minimal depression	18 (46.2)	31 (51.7)		**0.078**
Moderate depression	10 (25.6)	10 (16.7)		
Severe depression	2 (5.1)	6 (10)		
**ASEX-assessed sexual dysfunction, *n* (%)**			.179^b^	**0.161**
Absent	28 (71.8)	51 (85)		
Present	11 (28.2)	9 (15)		

Abbreviations: Quality of life (EUROHIS-QOL 8-Item Index); Depression (Beck Depression Inventory, BDI); Sexual function (Arizona Sexual Experiences Scale, ASEX); MLWH, men living with HIV; *r*, effect size for Mann–Whitney *U* test; φ (phi) or Cramer’s *V*, effect size for chi-square tests.

^a^Mann–Whitney *U* test.

**^b^**Chi-square test.

Bold values indicate statistically significant correlations (*p* < .05).

### 3.3. Correlations among age, quality of life, depression, and sexual function

**Figure 3.**
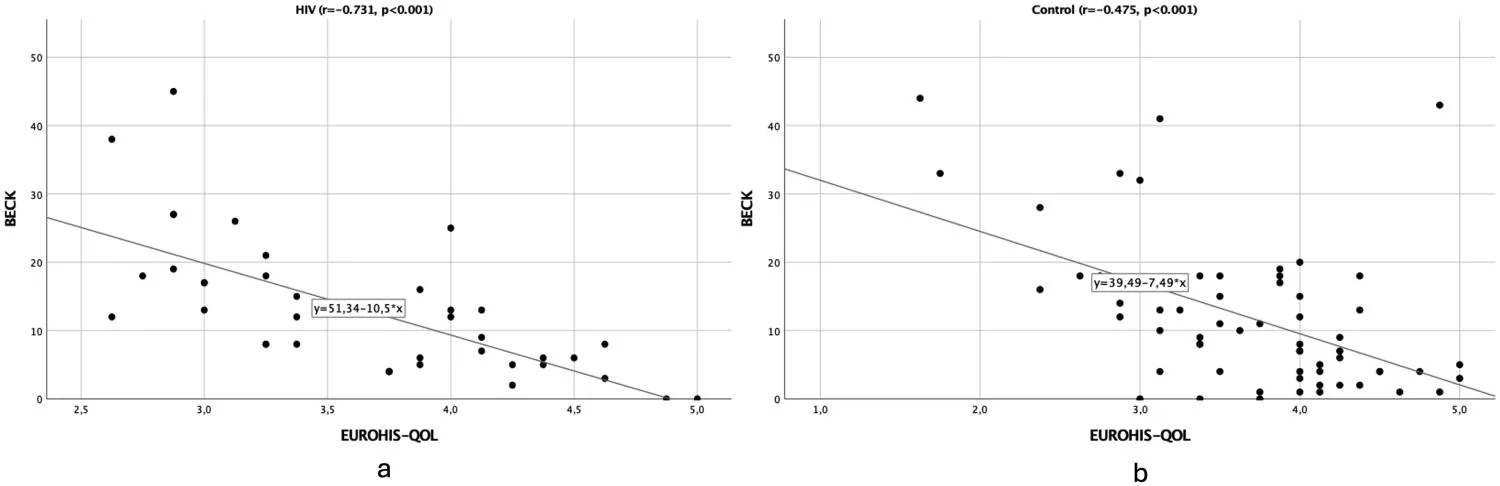
Scatter plots showing the correlation between quality of life (EUROHIS-QOL8) and depression (BDI) scores in men living with (a) HIV and (b) the controls.

In the MLWH, BDI scores exhibited a significant negative correlation with EUROHIS-QOL8 scores (*r* = –.731, *p* < .001), suggesting that increased depressive symptoms were associated with diminished quality of life. A similar but less significant negative correlation was observed in the control group (*r* = –.475, *p* < .001). In the control group, the EUROHIS-QOL8 scores exhibited an inverse correlation with the ASEX scores (*r* = –.396, *p* = .002), indicating that diminished sexual function was associated with a diminished quality of life (Table 3). Scatter plots illustrating these relationships are presented in Figure 3. In the HIV group, age was not significantly correlated with any psychometric variable. However, in the control group, age was positively correlated with the BDI (*r* = .502, *p* < .001) and negatively correlated with the EUROHIS-QOL8 (*r* = –.359, *p* = .005).

**Table 3.** Spearman’s rank correlations among age, depression, quality of life, and sexual function in men living with HIV and controls.

Variables	*r* (*p* value), MLWH (*n* = 39)	*r* (*p* value), control (*n* = 60)
Age vs. EUROHIS-QOL8	.001 (.996)	**–.359 (.005)**
Age vs. BDI	–.031 (.850)	**.502 (<.001)**
Age vs. ASEX	.048 (.773)	.154 (.239)
EUROHIS-QOL8 vs. BDI	**–.731 (<.001)**	**–.475 (<.001)**
EUROHIS-QOL8 vs. ASEX	–.290 (.073)	**–.396 (.002)**
BDI vs. ASEX	.298 (.065)	.217 (.097)

*Notes:* Higher BDI and ASEX scores indicate worse depression and greater sexual dysfunction, respectively, and a higher EUROHIS-QOL8 score indicates better quality of life.

Abbreviations: Quality of life (EUROHIS-QOL 8-Item Index); Depression (Beck Depression Inventory, BDI); Sexual function (Arizona Sexual Experiences Scale, ASEX); MLWH, men living with HIV; *r*, Spearman’s rank correlation coefficient.

Bold values indicate statistically significant correlations (*p* < .05).

## 4. Discussion

The present study evaluated the quality of life, depressive symptoms, and sexual function in MLWH compared with those in controls. The overall quality of life and depression scores were similar between the two groups. However, the ASEX scores were higher in the MLWH group, reflecting a modest difference in sexual function. There was a strong negative correlation between depression and quality of life in the HIV group, and a moderate negative correlation in the control group. These findings highlight the importance of addressing mental health as a key determinant of quality of life in both study groups.

Previous studies have reported a consistently high prevalence of depression among people living with HIV (PLWH), although the reported rates vary substantially by geographic region and study population. For example, data from the All of Us (AoU) Research Program in the United States showed that 43% of PLWH were diagnosed with major depressive disorder.^17^ Meanwhile, a pooled analysis from African cohorts estimated the prevalence of depressive symptoms among PLWH to be approximately 33.3%.^18^ These findings suggest that depression is prevalent among PLWH and is often reported at higher rates than in HIV-negative populations.

In contrast, no significant difference in depression scores was observed between the MLWH and healthy controls in the present study. One possible explanation for this is that our study population consisted exclusively of individuals with sustained virological suppression. Previous studies have shown that depressive symptoms tend to be more pronounced in people with poorer virological control, while lower symptom scores are observed in those who achieve viral suppression.^19^ Thus, effective antiretroviral therapy and stable virological control may have contributed to the comparable depression levels observed between the study groups.

In both groups, the presence of depressive symptoms was associated with lower quality of life. Previous studies, including those examining PLWH, have reported that depressive symptoms are independent factors associated with a reduced health-related quality of life.^20^^,^^21^ In our study, this association appeared stronger among MLWH. Overall, depressive symptoms remained clinically relevant in relation to quality of life in both study groups.

In this study, although ASEX scores were modestly higher among MLWH, the prevalence of sexual dysfunction defined by established ASEX cutoff criteria did not differ between groups.^11^ Previous studies have reported that sexual health in people living with HIV may be influenced by multiple biological and psychosocial factors, including emotional distress, reduced social support, HIV-related stigma, economic difficulties, and longer duration of antiretroviral therapy. Metabolic and vascular comorbidities, such as diabetes mellitus, dyslipidemia, and atherosclerosis, have also been associated with impaired sexual function.^22^^,^^23^ Although erectile dysfunction and other sexual problems have been reported more frequently among MLWH than in the general population, these conditions vary between individuals. For instance, a Brazilian study reported an erectile dysfunction prevalence of 37.5% among 120 MLWH, indicating that the majority of participants did not experience erectile dysfunction.^24^ Consistent with these observations, although no difference in the prevalence of sexual dysfunction was observed between groups in our study, the ASEX scores were significantly higher among the MLWH group. These findings support the importance of routinely addressing sexual health concerns in HIV care, even among virologically suppressed individuals.

In the present cohort, the median EUROHIS-QOL8 score was 3.75 for the PLWH group and 3.88 for the control group. In the Turkish population validated by Eser et al., the mean EUROHIS-QOL8 score was 3.46 ± 1.60.^14^ Our values were slightly higher overall. This difference may be due to the environmental factors. Our study was conducted in a province with higher income levels, better access to healthcare, and more social resources than other provinces. These advantages may have positively influenced the participants’ overall life satisfaction.

In the control group, age was correlated with moderately high levels of depression and a low quality of life. However, this trend was less apparent in the MLWH group. Previous studies have shown that depression is no more prevalent in middle-aged and older individuals with HIV than in HIV-negative individuals.^25^ HIV-related stressors and comorbidities may minimize the impact of age on depression. Additionally, the limited age range and small sample size of our study cohort may have reduced our ability to detect age-related associations with MLWH.

The strength of the study was that all participants were followed up at a comprehensive HIV center, ensuring consistent clinical observation and long-term patient-physician relationships. This fostering environment likely increased the accuracy and objectivity of survey responses.

This study has several limitations. First, although validated psychometric instruments were used, the assessments were based on self-reported perceptions rather than objective biological or hormonal measurements. Therefore, the findings reflect subjective experiences rather than direct physiological correlations. Second, the relatively small sample size may have limited the statistical power to detect subtle between-group differences. Third, the cross-sectional design prevents causal conclusions about the relationships between virological suppression, psychosocial factors, and patient-reported outcomes.

Future studies with larger sample sizes and longitudinal designs are needed to clarify how changes in treatment adherence, virological control, and psychosocial factors influence patient-reported outcomes over time. Including objective biological measures alongside patient-reported instruments may help interpret mental and sexual health outcomes in PLWH.

## 5. Conclusion

Overall, our findings suggest that, among MLWH who have achieved sustained virological suppression, depressive symptoms, quality of life, and prevalence of sexual dysfunction are similar to those in the general population. Although ASEX scores were modestly higher, this difference did not result in a higher prevalence of sexual dysfunction based on established criteria. These results support an individualized approach to care for MLWH based on their identified clinical needs rather than on assumptions related to their HIV status. Future research should include longitudinal studies to better understand how changes in adherence, virological control, and psychosocial factors interact over time.

## Data Availability

The authors will share the data and materials via email upon request. This study was conducted in accordance with the World Medical Association Declaration of Helsinki (2013 revision) and the protocols of the Non-Interventional Clinical Research Ethics Committee of the Kocaeli University Faculty of Medicine. The project was registered under number 2025/450 and received approval under code GOKAEK-2025/19/06 on 25 September 2025. This study was an anonymous survey that posed minimal risks. Verbal informed consent was obtained from all participants after they agreed to participate. They were informed that participation was voluntary and that they could withdraw from the study at any time.
